# Clinical Outcomes of Acellular Dermal Matrix (SimpliDerm and AlloDerm Ready-to-Use) in Immediate Breast Reconstruction

**DOI:** 10.7759/cureus.22371

**Published:** 2022-02-18

**Authors:** Brian P Tierney, Mauricio De La Garza, George R Jennings, Adam B Weinfeld

**Affiliations:** 1 Plastic Surgery, Tierney Plastic and Reconstructive Surgery, Nashville, USA; 2 Plastic and Reconstructive Surgery, DHR Health, McAllen, USA; 3 Plastic Surgery, Shoals Plastic Surgery Face and Body, Muscle Shoals, USA; 4 Surgery and Perioperative Care, Dell Medical School at the University of Texas, Austin, USA; 5 Plastic and Hand Surgery, Ascension Medical Group Seton, Austin, USA

**Keywords:** adm, alloderm, plastic and reconstructive surgery, integuply, breast cancer, simpliderm, extracellular matrix, complications, acellular dermal matrix, breast reconstruction

## Abstract

Background

The use of acellular dermal matrix (ADM) for post-mastectomy reconstruction is considered by many surgeons to be an accepted component of surgical technique. Early clinical experience is described for SimpliDerm® - a novel human ADM (Aziyo Biologics, Silver Spring, USA), and AlloDerm® Ready-To-Use (RTU) - an established ADM (Allergan Medical, Irvine, USA).

Methods

Records were retrospectively reviewed from four sites between 2016 and 2021 of patients who underwent immediate, two-stage reconstruction with either SimpliDerm (n=38) or AlloDerm RTU (n=69) after mastectomy and were followed out to exchange to permanent implant(s), tissue expander(s) explant, or death.

Results

Immediate breast reconstruction with tissue expanders and ADM was performed on 107 patients (181 breasts). Overall mean patient age was 51.4 ± 12.4 years, and mean BMI was 28.0 ± 5.8 kg/m^2^. Significantly more patients in the SimpliDerm group were of Hispanic or Latino ethnicity (34.2% vs. 7.2%; P<.001). Reconstructions were predominantly prepectoral (82.3%). A total of 35 adverse events (AEs) occurred in 27 (25.2%) patients, with no difference in AE type, classification, or rates between ADM groups. No AEs were considered related to either ADM. The observed AE profiles and rates are similar to those published for other ADMs in immediate breast reconstruction.

Conclusions

There continues to be a need for additional clinically equivalent ADMs to provide physicians with more availability and options for their practice. This retrospective, multisite study describes comparable clinical outcomes with SimpliDerm and AlloDerm RTU through a median of 133.5 days (~four months) following immediate two-stage breast reconstruction.

## Introduction

Acellular dermal matrices (ADMs) are used in approximately 60% of all breast reconstructions performed every year in the United States (US) [1]. In addition to the common rationale for employing ADMs in prosthetic breast reconstruction (providing stabilization of the implant or tissue expander, support of inframammary fold and the lower pole, eliminating the need for donor site morbidity, coverage for the prepectoral implant placements), ADMs have favorably been reported to reduce capsular contracture risk, improve cosmetic outcomes, and allow for greater intraoperative fill volumes in two-stage expander-based reconstructions [2-8]. In prospective and retrospective clinical trials, commercially available ADMs have demonstrated roughly comparable complication rates in both submuscular and prepectoral breast reconstructions [2, 9-11]. However, controversy remains with the use of ADM in breast reconstructive procedures [11], therefore, there continues to be a need for additional safety and efficacy data of available ADMs to provide greater accessibility, more physician choice, increased payer coverage, and to foster ADM innovation through improved design of newer products [9, 12]. In an effort to support the existing literature and introduce additional data on a new human ADM, we report a collective experience with two ADMs in immediate breast reconstruction (IBR).

ADMs are manufactured from decellularized biologic xenograft or allograft material. In addition to their basic clinical characteristics and species/tissue source (eg, human cadaveric dermis, hADM, is used in our practices), ADMs may be differentiated based on their processing methods during manufacturing. The different manufacturing processes required to remove immunogenic components and sterilize the matrix give rise to a variety of ADMs with differing physical and biological characteristics. Different processing methods may affect product handling, remodeling, and integration into native tissues, which could influence clinical performance and overall cost (eg, price, reoperations, complication management, etc.).

Enhanced preservation of extracellular matrix (ECM) structure has been shown in clinical and pre-clinical studies to support the integration of ADM into native tissues by promoting constructive tissue remodeling processes such as neovascularization and inflammation [13, 14]. SimpliDerm® (Aziyo Biologics, Silver Spring, USA) is a novel ADM manufactured from human dermis using patented methods designed to preserve the natural ECM structure and intrinsically active biologic factors needed to support tissue remodeling. Preclinical study results in a comparative, clinically relevant non-human primate model suggest that SimpliDerm may be biologically superior to an established hADM (AlloDerm® ready-to-use (RTU), Allergan Medical, Irvine, USA) in the host response to the implant. This comparison study found that SimpliDerm degraded more gradually, caused a lesser initial inflammatory reaction, and expressed less pro-fibrotic markers and proinflammatory cytokines compared to AlloDerm RTU [15]. The observed differences in the pre-clinical host response to these hADMs suggest that the use of SimpliDerm leads to a reduced inflammatory and fibrotic response, and improved remodeling compared to AlloDerm RTU.

A recent publication reports early (30-day) outcomes of a retrospective series using two ADMs (SimpliDerm and AlloDerm RTU) in immediate, two-stage breast reconstructions [16]. However, the most dynamic period of reconstructions occurs throughout the tissue expansion phase, so understanding ADM performance through this entire period is clinically important. This report focuses on the safety and efficacy outcomes of the same two ADMs but in a larger, multi-center patient population with follow-up through the implant exchange procedure.

## Materials and methods

Each participating site received approval from a review board (Western Institutional Review Board, WIRB) to perform a retrospective chart review of SimpliDerm and AlloDerm RTU used in consecutive patients undergoing immediate, two-stage breast reconstruction post-mastectomy between August 2016 and July 2021. Patients were excluded if they underwent delayed breast reconstruction, cosmetic or aesthetic breast augmentation or revision, or revision of a prior breast reconstruction.

**Figure 1:**
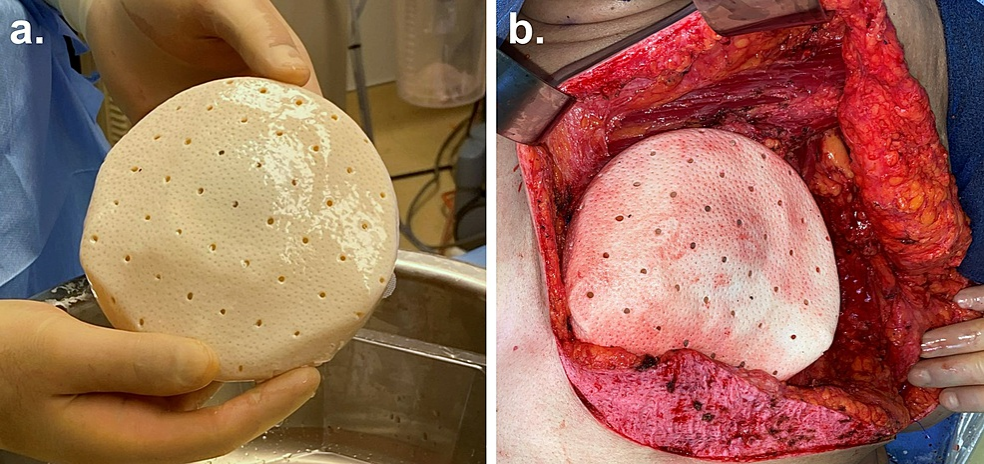
Intraoperative photos of SimpliDerm Hydrated Acellular Dermal Matrix SimpliDerm Hydrated Acellular Dermal Matrix (ADM) comes pre-hydrated and ready-to-use: (a) a perforated, rectangular piece of SimpliDerm ADM wrapped around a tissue expander, and (b) the ADM-expander construct being placed within the tissue pocket.

All treating surgeons were experienced with using both hADMs in ADM-based breast reconstruction, and their general surgical technique and postoperative protocol was similar between surgeons and hADM products. Both SimpliDerm and AlloDerm RTU were prepared based on the appropriate manufacturer’s instructions and used to cover the tissue expander (Figure 1). The choice of hADM in each procedure was based on facility availability at the time of surgery for all physicians. As described in previous publications, the ADM was sutured to the inframammary fold and pectoralis major muscle [2, 6, 7]. Two drains were placed intraoperatively as previously described: one removed postoperatively after one week, and the other removed after output volume was less than 30 mL in a 24-hour period (normally performed at the two-week post-op visit in surgeons’ standard care) [16]. At week three, patients typically began tissue expansion.

Standard patient-specific and procedural data were extracted from the medical records along with peri- and postoperative outcomes and complications. Complications related to surgical procedure or disease were reported during the perioperative period and recorded as requiring or not requiring explantation and/or surgical intervention. Adverse events were recorded as any untoward medical occurrence in a study subject that occurred at any time during follow-up and may or may not have been related to the study intervention. Serious adverse events were those considered to be life-threatening, resulting in death, requiring hospitalization, resulting in significant disability, or requiring intervention to prevent these outcomes. Major complications were defined as those requiring hospitalization and/or surgical intervention, and minor complications as those requiring outpatient clinic treatment only [9].

Statistical analysis

Continuous variables were assessed for normality. Independent samples t-tests were used to compare mean differences between cohorts. Medians were compared using Mann Whitney U test. Pearson chi-squares were reported for comparisons with expected cell counts of five or greater, and Fisher’s exact tests were reported if a cell count was less than five. Statistical significance was set to P<.05. SPSS version 26 (IBM Corp., Armonk, USA) was used for statistical analysis.

## Results

Records from four centers in the US totaling 107 patients (181 breasts) who underwent immediate, two-stage, tissue-expander-based breast reconstruction with the use of SimpliDerm (38 patients, 67 breasts) or AlloDerm RTU (69 patients, 114 breasts), followed by exchange to permanent implant(s), tissue expander(s) explant, or death were reviewed during the study period.

Patient demographics

Patient demographics are listed in Table 1 alongside preoperative comorbidities. The average age of all patients was 51.4 ± 12.4 years, and most patients were white and non-Hispanic or Latino (83.2%). Comorbid medical history included overweight (31.8%) and obese (32.7%) individuals, those who were current (1.9%) or former (15.0%) smokers, and those with diabetes (11.2%), hypertension (24.3%), and/or hypercholesterolemia (13.1%). Most patients (88.8%) had a previous or current cancer diagnosis, and 93.5% did not receive pretreatment cancer medication, anticoagulants, antibiotics, or pain medication. The majority of patients did not receive pretreatment chemotherapy or radiation (83.2%), but those that did (16.8%) underwent either chemotherapy or chemotherapy and radiation.

**Table 1:** Patient demographics * P-value is SimpliDerm vs. AlloDerm RTU ¥ Fisher’s Exact test used instead of Pearson Chi-Square b/c at least 1 expected cell count < 5. RTU: ready-to-use

	All Patients	SimpliDerm	AlloDerm RTU	P-value*
	(N=107)	(n=38)	(n=69)	
Age (years), mean ± SD	51.4 ± 12.4	48.8 ± 11.4	52.8 ± 12.9	0.111
Race				0.268
White	98 (91.6%)	33 (86.8%)	65 (94.2%)	--
Black or African American	7 (6.5%)	3 (7.9%)	4 (5.8%)	--
Other	2 (1.9%)	2 (5.3%)	0 (0.0%)	--
Ethnicity				<0.001
Non-Hispanic or Latino	89 (83.2%)	25 (65.8%)	64 (92.8%)	--
Hispanic or Latino	18 (16.8%)	13 (34.2%)	5 (7.2%)	--
BMI (kg/m^2^), mean ± SD	28.0 ± 5.8	28.6 ± 5.2	27.6 ± 6.2	0.399
BMI category (kg/m^2^)				0.622
Underweight (<18.5)	2 (1.9%)	0 (0.0%)	2 (2.9%)	--
Normal (18.5 to <25.0)	36 (33.6%)	11 (28.9%)	25 (36.2%)	--
Overweight (25.0 to <30.0)	34 (31.8%)	13 (34.2%)	21 (30.4%)	--
Obesity, Class I-II (30.0 to <40.0)	34 (31.8%)	14 (36.8%)	20 (29.0%)	--
Obesity, Class III (≥40.0)	1 (0.9%)	0 (0.0%)	1 (1.4%)	--
Smoking				0.259
Never	87 (81.3%)	29 (76.3%)	58 (84.1%)	--
Former	16 (15.0%)	6 (15.8%)	10 (14.5%)	--
Current	2 (1.9%)	2 (5.3%)	0 (0.0%)	--
Unknown	2 (1.9%)	1 (2.6%)	1 (1.4%)	--
Medical history				--
Previous or current cancer diagnosis	95 (88.8%)	34 (89.5%)	61 (88.4%)	1.000^¥^
Diabetes (type 1 or 2)	12 (11.2%)	3 (7.9%)	9 (13.0%)	0.533^¥^
Hypertension	26 (24.3%)	9 (23.7%)	17 (24.6%)	0.912
Hypercholesterolemia	14 (13.1%)	4 (10.5%)	10 (14.5%)	0.766
Pretreatment medication type				0.490
Cancer treatment medication	5 (4.7%)	3 (7.9%)	2 (2.9%)	--
Anticoagulant	0 (0.0%)	0 (0.0%)	0 (0.0%)	--
Antibiotic	1 (0.9%)	0 (0.0%)	1 (1.4%)	--
Pain Medication	1 (0.9%)	0 (0.0%)	1 (1.4%)	--
None	100 (93.5%)	35 (92.1%)	65 (94.2%)	--
Pretreatment chemotherapy or radiation (RTx)				0.051
No	89 (83.2%)	28 (73.7%)	61 (88.4%)	--
Yes	18 (16.8%)	10 (26.3%)	8 (11.6%)	--
Chemotherapy	15 (83.3%)	9 (90.0%)	6 (75.0%)	--
Chemotherapy and RTx	3 (16.7%)	1 (10.0%)	2 (25.0%)	--

Relating to ADM types, there were no statistically significant differences between groups for patient age, race, BMI, smoking status, medical history, or pretreatment medications (Table 1). Regarding ethnicity, there was a significantly higher proportion of Hispanic or Latino patients in the SimpliDerm group (34.2% SimpliDerm vs. 7.2% AlloDerm RTU; P<.001). Differences in rates of pretreatment chemotherapy and/or radiotherapy were of borderline significance, with a higher proportion of patients in the SimpliDerm group receiving pretreatment chemotherapy (26.3% vs. 11.6%; P=.051).

Procedural details

Mastectomy indication was predominantly for the treatment of malignancy (87.3%) (Table 2). The plane of most implantations was prepectoral (82.3%), however, some (17.7%) were performed in the subpectoral plane. Most procedures were bilateral (81.8%) and approximately half of the mastectomies used skin-sparing (49.2%) and the other half nipple-sparing (50.3%) techniques. The average intraoperative expander fill volume was 327.7 ± 167.0 mL.

**Table 2:** Procedural details (by breast) * P-value is SimpliDerm vs. AlloDerm RTU hADM: human cadaveric acellular dermal matrix; RTU: ready-to-use

	All Patients	SimpliDerm	AlloDerm RTU	P-value*
	(N=181)	(n=67)	(n=114)	
Plane of expander/implant placement				<0.001
Prepectoral	149 (82.3%)	67 (100.0%)	82 (71.9%)	--
Subpectoral	32 (17.7%)	0 (0.0%)	32 (28.1%)	--
Intraoperative expander fill volume (mL), mean ± SD	327.7 ± 167.0	358.6 ± 191.3	309.5 ± 147.9	0.074
hADM perforated (versus not)				0.121
Yes	76 (42.0%)	26 (38.8%)	50 (43.9%)	--
Unknown	26 (14.4%)	6 (9.0%)	20 (17.5%)	--
Mastectomy Type				0.600
Skin-sparing	89 (49.2%)	31 (46.3%)	58 (50.9%)	--
Nipple-sparing	91 (50.3%)	36 (53.7%)	55 (48.2%)	--
Simple Mastectomy	1 (0.6%)	0 (0.0%)	1 (0.9%)	--
Bilateral or Unilateral				0.396
Bilateral	148 (81.8%)	58 (86.6%)	90 (78.9%)	--
Unilateral – left	17 (9.4%)	4 (6.0%)	13 (11.4%)	--
Unilateral – right	16 (8.8%)	5 (7.5%)	11 (9.6%)	--
Mastectomy Indication				0.812
Malignancy (therapeutic)	158 (87.3%)	59 (88.1%)	99 (86.8%)	--
Prophylactic	23 (12.7%)	8 (11.9%)	15 (13.2%)	--

The bilateral reconstruction rate did not differ by ADM type (86.6% SimpliDerm, 78.9% AlloDerm RTU; P=.396) (Table 2). Use of perforated hADM, mastectomy type (skin-sparing vs. nipple-sparing), and mastectomy indication (therapeutic vs. prophylactic) did not differ between groups. While the majority of reconstructions in both groups used a prepectoral implant placement, significantly more patients in the AlloDerm RTU group had subpectoral placement (28.1% vs. 0%; P<.001), whereas all patients in the SimpliDerm group had prepectoral placements. There was a numeric trend toward greater intraoperative expander fill volumes in the SimpliDerm group (358.6 cc SimpliDerm vs. 309.5 cc AlloDerm RTU), but this difference did not quite reach statistical significance (P=.074). 

Follow-up and complications

Follow-up details and reported complications are listed in Table 3. Patients were followed until exchange to permanent implant(s), tissue expander(s) explant, or death, with a median follow-up time of 133.5 days (~4 months). The median time to last drain removal was 15 days, and most patients were on pain medications (93.5%) and/or antibiotics (80.4%) during one or more follow-up visits. The median time to the exchange procedure was 128 days; some patients (16.8%) underwent post-treatment chemotherapy and/or radiation during this time. The average final expander fill volume was 487.6 ± 154.8 mL and the permanent implant fill volume was 545.4 ± 149.6 mL (Table 4). Complications resulting in surgical intervention were low (21.5%), with even lower rates of complications leading to explantation (11.2%).

**Table 3:** Follow up and Complications (by patient) One Patient in the AlloDerm cohort had both an explant (right side) and exchange procedure (left side). This patient is counted in each variable. Medians were compared using Mann Whitney U Test * P-value is SimpliDerm vs. AlloDerm RTU ¥ Fisher’s Exact test used instead of Pearson Chi-Square b/c at least 1 expected cell count < 5. RTU: ready-to-use

	All Patients	SimpliDerm	AlloDerm RTU	P value*
	(N=107)	(n=38)	(n=69)	
Follow up time in days, median (25^th ^– 75^th^ percentile)	133.5 (98 – 223)	123.5 (85.5 – 188)	144.5 (101 – 256.5)	0.077
Days to last drain removal, median (25^th ^– 75^th^ percentile)	15 (14 - 20.3)	15 (14 – 18)	15 (14 – 21.8)	0.291
Current medications (during ≥1 follow-up)				--
Pain medication	100 (93.5%)	37 (97.4%)	63 (91.3%)	0.417^¥^
Anticoagulants	4 (3.7%)	2 (5.3%)	2 (2.9%)	0.614^¥^
Antibiotics	86 (80.4%)	33 (86.8%)	53 (76.8%)	0.211
None	2 (1.9%)	1 (2.6%)	1 (1.4%)	1.000^¥^
Chemotherapy or radiotherapy (RTx) during follow-up				0.943
Chemotherapy	9 (8.4%)	3 (7.9%)	6 (8.7%)	--
RTx	7 (6.5%)	3 (7.9%)	4 (5.8%)	--
Chemotherapy and RTx	2 (1.9%)	1 (2.6%)	1 (1.4%)	--
Post-mastectomy complications	N=54	n=20	n=34	--
Flap ischemia	10 (9.3%)	3 (7.9%)	7 (10.1%)	1.000^¥^
Dehiscence	2 (1.9%)	1 (2.6%)	1 (1.4%)	1.000^¥^
Hematoma	2 (1.9%)	0 (0.0%)	2 (2.9%)	0.538^¥^
Seroma	9 (8.4%)	4 (10.5%)	5 (7.2%)	0.718^¥^
Red breast syndrome	3 (2.8%)	1 (2.6%)	2 (2.9%)	1.000^¥^
Infection	8 (7.5%)	4 (10.5%)	4 (5.8%)	0.451^¥^
Capsular contracture	2 (1.9%)	0 (0.0%)	2 (2.9%)	0.538^¥^
Death of unknown cause	1 (0.9%)	0 (0.0%)	1 (1.4%)	1.000^¥^
Other	17 (15.9%)	7 (18.4%)	10 (14.5%)	0.595
Complication(s) resulting in surgical intervention, n (%)	23 (21.5%)	10 (26.3%)	13 (18.8%)	0.368
Complication(s) resulting in explantation, n (%)	12 (11.2%)	6 (15.8%)	6 (8.7%)	0.340^¥^
Days to exchange procedure, median (25^th^- 75^th^ percentile)	128 (99.3 – 176.8)	127.5 (98 – 200.3)	131 (103.8 – 176.3)	0.557

Median follow-up time was similar between groups (123.5 days SimpliDerm vs. 144.5 days AlloDerm RTU; P=.077), trending toward longer follow-up time in the AlloDerm RTU group (Table 3). There were no statistically significant differences between groups during follow-up in the use of pain medication, anticoagulants, or antibiotics; treatment with chemotherapy or radiotherapy; or incidence of postmastectomy complications. Time to last drain removal was equivalent in both groups (15 days; P=.291).

Throughout the follow-up period, 60.5% of the SimpliDerm group and 68.1% of the AlloDerm RTU group remained free of complications (P=.430). There were no differences between groups in the proportion of patients who reached their exchange procedure (84.2% SimpliDerm vs. 91.3% AlloDerm RTU; P=.340) or in the number of days from reconstruction to exchange. The proportion of patients with complications requiring surgical intervention or resulting in explantation also did not differ between groups - a total of six patients in each group had a complication that resulted in explantation (15.8% SimpliDerm vs. 8.7% AlloDerm RTU; P=.340). Final expander fill volumes and permanent implant volumes for patients who underwent an exchange procedure (N=162 breasts) also were comparable between groups (Table 4).

**Table 4:** Fill volumes (by breast in patients with exchange procedure, N=162†) * P-value is SimpliDerm vs. AlloDerm RTU † Five patients were missing final expander fill volume data RTU: ready-to-use

	All Patients	SimpliDerm	AlloDerm RTU	P-value*
	(N=156)	(n=54)	(n=102)	
Final expander fill volume in mL, mean ± SD	487.6 ± 154.8	504.0 ± 116.8	478.9 ± 170.3	0.281
	(N=160)	(n=56)	(n=104)	
Permanent implant fill volume in mL, mean ± SD	545.4 ± 149.6	545.8 ± 121.2	545.2 ± 161.3	0.978

Adverse events

A total of 35 postoperative adverse events (AEs) were reported in 27 (25.2%) patients (Table 5). Most AEs were considered not serious (62.9%). The most common AEs were infection (22.9%), flap ischemia (25.7%), and seroma (14.3%). Management actions (medication, procedure, or other) were used for most reported AEs (94.9%). There were no differences between ADM groups in AE rates; the seriousness, classification, or type of AE; or in actions taken to manage AEs.

**Table 5:** Postoperative adverse events (total of 35 AEs in 27 patients) Values are shown as n (% of AEs) A serious adverse event was defined as an event that 1) threatened life, 2) resulted in permanent impairment of a body function or permanent damage to a body structure, 3) necessitated medical or surgical intervention to preclude such impairment, 4) required or prolonged hospitalization, or 5) was fatal. * P-value is SimpliDerm vs. AlloDerm RTU † Major complications were those requiring hospitalization and/or surgical intervention. Minor complications required outpatient clinic treatment only [9]. ‡ Two AEs had two actions taken and one AE had three actions taken. All other AEs had one action taken. RTU: ready-to-use; AE: adverse event; Pt.: patient

	All Patients	SimpliDerm	AlloDerm RTU	P-value*
	(35 AE in 27 Pt.)	(18 AE in 14 Pt.)	(17 AE in 13 Pt.)	
Adverse event seriousness				0.631
Not serious	22 (62.9%)	12 (66.7%)	10 (58.8%)	--
Serious	13 (37.1%)	6 (33.3%)	7 (41.2%)	--
AE classification†				0.601
Minor	16 (45.7%)	9 (50.0%)	7 (41.2%)	
Major	19 (54.3%)	9 (50.0%)	10 (58.8%)	
AE type				0.519
Infection	8 (22.9%)	4 (22.2%)	4 (23.5%)	--
Flap ischemia	9 (25.7%)	3 (16.7%)	6 (35.3%)	--
Hematoma	1 (2.9%)	0 (0.0%)	1 (5.9%)	--
Seroma	5 (14.3%)	3 (16.7%)	2 (11.8%)	--
Dehiscence	1 (2.9%)	1 (5.6%)	0 (0.0%)	--
Red breast syndrome	1 (2.9%)	1 (5.6%)	0 (0.0%)	--
Death of unknown cause	1 (2.9%)	0 (0.0%)	1 (5.9%)	--
Other	9 (25.7%)	6 (33.3%)	3 (17.6%)	--
Action taken‡				0.426
Medication	8 (20.5%)	4 (19.0%)	4 (22.2%)	--
Procedure	26 (66.7%)	13 (61.9%)	13 (72.2%)	--
None	2 (5.1%)	1 (4.8%)	1 (5.6%)	--
Other	3 (7.7%)	3 (14.3%)	0 (0.0%)	--

## Discussion

Studies have demonstrated multiple benefits of using ADM in breast reconstruction, including subpectoral and prepectoral implant placements [2, 6, 9-11]. Despite recent publications of randomized trials comparing different ADMs in breast reconstruction, no individual product has yet demonstrated superiority to other ADMs [2, 9, 10, 17, 18]. As production of each commercially-available ADM is limited by access to human donor tissue, there continues to be a need for additional ADMs that yield equivalent clinical outcomes to provide more availability and options for physicians. Initial 30-day outcomes from a single surgeon’s experience with both SimpliDerm and AlloDerm RTU were previously reported, finding that both ADMs perform equivalently during that time period [16]. The data in this report provides similar findings regarding the utility and safety of the new hADM SimpliDerm as a clinically equivalent alternative to AlloDerm RTU in immediate, two-stage breast reconstruction, and includes follow-up through the critical post-reconstructive phase through to exchange procedure.

While most ADMs appear to perform equivalently in the clinical setting, there are differences in their intraoperative preparation, physical characteristics, and storage conditions related to the method of processing [13]. Both ADMs used in this clinical study are derived from human dermal tissue and are packaged hydrated and “ready-to-use”, yet SimpliDerm and AlloDerm RTU differ in their methods of processing. Per AlloDerm’s Instructions For Use (IFU), electron beam irradiation is used to sterilize AlloDerm RTU to a Sterility Assurance Level (SAL) of 10^-3^ whereas SimpliDerm’s IFU states that it is terminally sterilized to a SAL of 10^-6^ using a proprietary ionizing radiation system. While these processing methods may not affect extracellular matrix stability, the divergent sterility levels could affect microbial colonization vulnerability [15]. Other potential benefits of the proprietary processing methods of SimpliDerm have been elucidated in rigorous preclinical studies. For example, a non-human primate model demonstrated that SimpliDerm facilitates host responses that foster remodeling of the hADM into native tissue [15]. In that study, SimpliDerm demonstrated a milder initial inflammation, more gradual implant degradation, reduced expression of profibrotic genes, and reduced long-term inflammatory markers compared to the host response to AlloDerm RTU. In the clinical experience reported here, no discernible difference was seen between clinical outcomes of either ADM. However, there are differing surgical handling and physical characteristics that we have observed while using both hADMs. We believe SimpliDerm has superior handling and pliability, the reported thickness was more consistent across each individual piece, and the actual lengths/widths were more reliable based on chosen ADM size compared to equivalently sized pieces of AlloDerm RTU.

The total adverse event rate in our dataset was 25.2% - consistent with rates reported in the literature, which range from 3.9%-33.5% following ADM-based reconstructions [2-8, 12, 19, 20]. Importantly, the incidence of unplanned hADM explantation in this study was low (11.2%) with no difference between ADM type, and mirrors rates reported in the literature: a meta-analysis of 21 studies of ADM-assisted breast reconstructions reported ADM explanation rates ranging from 1.3% - 23.8% [20]. The most common individual complications in this study - infection (7.5%), flap ischemia (9.3%), and seroma (8.4%) - were also consistent with rates reported in published studies [2-8, 12, 19, 20].

Through numerous clinical studies, AlloDerm has demonstrated similar outcomes to many other ADMs used in breast reconstructions [2, 4, 9, 10, 17, 18]. These commercially available pre-hydrated ADMs are also derived from human dermis and include Cortiva® (RTI Surgical, Alachua, USA), DermACELL® (LifeNet Health, Virginia Beach, USA), and FlexHD® (MTF, Edison, USA). In our study, adverse event rates were not significantly different between SimpliDerm and AlloDerm RTU groups (12.2% vs. 13.1%, respectively) and there was no difference in AE classification between groups (P=.601). While there were numeric differences in complication rates between the two ADMs, none were statistically significant. Nevertheless, factors that could contribute to numeric trends in infection and seroma rates might include corresponding trends toward greater intraoperative expander fill volumes with SimpliDerm (358.6 vs. 309.5 mL; P=.074) and the slightly lower use of perforated ADM with SimpliDerm (38.8% vs. 43.9%; P=.121). Although ADM use in two-stage breast reconstruction can allow for greater initial expander fill volumes compared to no ADM [21], some studies have reported a significant association between higher initial fill volumes and postoperative complications, including infection, explantation, flap necrosis, and others [22, 23]. One study found that the risk of explantation increased two-fold if intraoperative expander fill volume surpassed 300 mL [23]. Fenestration of ADM has been associated with reduced risk for seroma formation in some studies, though not in others [24, 25]. These associations remain speculative and all require further investigation.

Pretreatment chemotherapy or radiation therapy

Published evidence is conflicting regarding the impact of neoadjuvant cancer therapies on the outcomes of breast reconstruction. Data from several studies describe a significant association between radiation therapy and postoperative complications [22, 23, 26]. However, an analysis of a randomized trial did not find significant associations between pretreatment chemotherapy or radiotherapy and risk for complications [9].

The patients in this dataset who underwent pretreatment chemotherapy and/or radiation therapy (16.8%) were similar to the number of patients in previously published analyses (10.6% - 33.3%) which described significant associations with postoperative complications [22, 23, 26]. A larger proportion of patients in the SimpliDerm group had premastectomy chemotherapy and/or radiotherapy, a difference of borderline significance (26.3% vs. 11.6%; P=.051). Whether higher rates of chemotherapy in the SimpliDerm group affected rates of adverse events in this group remains an open question and merits further investigation.

Ethnicity and outcomes of breast reconstruction

In general, ethnicity is under-reported in ADM-based breast reconstruction publications, and the relationship of ethnicity to access to care and clinical outcomes can be complex. Many studies have reported lower rates of breast reconstruction and higher rates of adverse events following reconstruction in Hispanic and Latino patients compared to non-Hispanic whites [27-30]. The SimpliDerm group enrolled a significantly larger proportion of Hispanic or Latino patients compared to the AlloDerm RTU group (34.2% vs. 7.2%; P<.001). In the current study, SimpliDerm was associated with similar rates of complications and adverse events to AlloDerm RTU, despite a higher proportion of Hispanic or Latino patients. When we stratified the complication rates of Hispanic and Latino patients, we observed an increased rate of complications in the cohorts vs. the two cohorts at-large (61.5% vs. 52.6% SimpliDerm and 100.0% vs. 49.3% AlloDerm RTU, respectively). However, interpreting this finding is challenging in the absence of clear trends in ADM breast reconstruction literature and the small patient sample of Hispanic and Latino patients in our dataset. Future studies of ADM-based breast reconstruction should aim to report the ethnic profile of their patient populations and potentially investigate outcomes by ethnicity to better understand issues related to disparities in care and surgical outcomes.

Limitations

This study was limited by its retrospective and non-randomized design, the number of patients, mixed plane of expander/implant placement, and length of follow-up. The non-randomized design makes it difficult to understand if patient selection played a role in the over-representation of individuals with specific characteristics, such as pretreatment chemotherapy or Hispanic or Latino ethnicity, or how these characteristics may have affected outcomes. Also, since our ethnicity data were gathered retrospectively from existing patient charts, the accuracy of this data is unknown. The modest sample size may have prevented the identification of statistical significance in outcomes that showed numerical trends. Additional studies are warranted to determine clinical outcomes between sub- and pre-pectoral placement in patients receiving these ADMs, as this was not possible with our small cohort. Studies reporting long-term clinical outcomes will further our understanding of these ADMs and their long-term complication profiles. These limitations, while frequent in recently published studies due to intrinsic challenges related to studying ADMs in post-mastectomy breast reconstruction, nevertheless limit the interpretability of study findings. The longer-term (post-exchange procedure) consequences of post-mastectomy breast reconstruction remain an additional clinical concern and need to be addressed in future investigations.

## Conclusions

Implant-based breast reconstruction will continue to be performed in conjunction with ADM, as more studies continue to demonstrate the safety and clinical and aesthetic benefits in this setting. Our findings support that SimpliDerm is a clinically equivalent ADM to AlloDerm RTU that is safe and effective for two-stage breast reconstructions through over four months of follow-up.
